# Supplementary material for: Discovery of novel astrovirus genotype species in small ruminants
Source: PeerJ. 2019 Jul 31;7:e7338. doi: 10.7717/peerj.7338 (PMC6679648; doi:10.7717/peerj.7338)
Supplement: Supplemental Information 1 [file peerj-07-7338-s001.docx]

**Supplementary table 1: Primer and probe sequences**

Detection of neurotropic astroviruses BoAstV-CH13 & ovAstV-CH16

| Name | Primer/Probe Sequence ( 5' - 3' ) ^a^ | Target | Reference |
| --- | --- | --- | --- |
| CH13-A_Forward  CH13-A_Reverse  CH13-A_Probe | AGGCATGACTATGAGCGCGT  AATCCGGTTGTGCCACCTCA  ^FAM^GGCAACGCACAGGCACTTG^BHQ1^ | BoAstV-CH13: Part of 5' end of ORF1a | Lüthi R, Boujon CL, Kauer R, Koch MC, Bouzalas IG, and Seuberlich T. 2018. Accurate and precise real-time RT-PCR assays for the identification of astrovirus associated encephalitis in cattle. Scientific Reports 8:9215. 10.1038/s41598-018-27533-8 |
| CH13-B_Forward  CH13-B_Reverse  CH13-B_Probe | TTTTGGCTCGTCACTTTGTG  ACAACCTCCTTGGCAATCTG  ^FAM^GATAAGCTTTGGAGGGGAGG^BHQ1^ | BoAstV-CH13: Center of ORF2 |  |
| CH16_Forward  CH16_Reverse  CH16_Probe | CGTAGCACCCCCTTACAGCA  CCTCGATCCTACTCGGCGTG  ^FAM^CTTAGAGGCCACGCAGAAGC^BHQ1^ | OvAstV-CH16: 3' end of ORF2 | Kuchler L, Koch MC, Seuberlich T, and Boujon CL. 2019. Archive Mining Brings to Light a 25-Year Old Astrovirus Encephalitis Case in a Sheep. Front Vet Sci 6:51. 10.3389/fvets.2019.00051 |

^a^FAM, 6-Carboxyfluorescein; BHQ1, Black Hole Quencher 1

Detection of various astroviruses using PanAstV-RT-PCR

| Name | Primer/Probe Sequence ( 5' - 3' ) ^a^ | Target | Reference |
| --- | --- | --- | --- |
| PanAstV_Forward1  PanAstV_Forward2  PanAstV_Reverse  PanAstV_Forward_nested1  PanAstV_Forward_nested2 | GARTTYGATTGGRCKCGKTAYGA  GARTTYGATTGGRCKAGGTAYGA  GGYTTKACCCACATNCCRAA  CGKTAYGATGGKACKATHCC  AGGTAYGATGGKACKATHCC | Various AstV: 3' end of ORF1b | Chu DK, Chin AW, Smith GJ, Chan KH, Guan Y, Peiris JS, and Poon LL. 2010. Detection of novel astroviruses in urban brown rats and previously known astroviruses in humans. J Gen Virol 91:2457-2462. 10.1099/vir.0.022764-0 |

| Name | Primer/Probe Sequence ( 5' - 3' ) ^a^ | Target |
| --- | --- | --- |
| CapAstV-G2.1_500R  CapAstV-G2.1_301R | GTGATGTTAGGACCGCGTCA  AGTGGGCCAACTGAGTGTAA | 5' end position as indicated in primer-annotation |
| CapAstV-G3.1_508R  CapAstV-G3.1_320R | ACGAATAAGGCATGACCGCA  AAAGCCTGTGATCCATGCCA |  |
| CapAstV-G5.1_519R  CapAstV-G5.1_325R | TCCGTTGTGGAAGTGGTTGT  GGCACGATCATTCATCAGCG |  |
| OvAstV-S5.1_518R  OvAstV-S5.1_325R | CTGTCTTCATGGCGTTCTCA  ATTACGGTGTTGGGCCAGTT |  |
| OvAstV-S6.1_501R  OvAstV-S6.1_309R | TGTATGATGGGTGTGGACTGT  AGTGGTGCTCCTCTCTTCAT |  |

Rapid amplification of cDNA-ends

| Name | Primer/Probe Sequence ( 5' - 3' ) ^a^ | Target |
| --- | --- | --- |
| G2.1_F  G2.1_R  G2.1_P | GGGCCAGCATGAAATACACCAG  AGGGATGACCAGCTGACTTGAG  ^FAM^CCGAGTGTCAGCCGTTCCTA ^BHQ1^ | Position 4'006 to 4'305 (ORF2) |
| G3.1_F  G3.1_R  G3.1_P | CCAACACAAGTGTCGTGGTCAG  CAAGTTCATTAGGCCAGGCTGC  ^FAM^AACACTCTGACACCACCCCG ^BHQ1^ | Position 4'219 to 4'518 (ORF2) |
| G5.1_F  G5.1_R  G5.1_P | CAACCACGCTGGATAATGGTGG  AGGCATTTTGAGGGGTCCTGAT  ^FAM^CCAGGCACAGGCTCAGATCA ^BHQ1^ | Position 5'385 to 5'684 (ORF2) |
| S5.1_F  S5.1_R  S5.1_P | CCTTGCATTGCCTGTTGGAGAG  TGTGCCTGGGTGTATGATGGTC  ^FAM^CCAACGCGAGGTATCCCTGT ^BHQ1^ | Position 5'155 to 5'454 (ORF2) |
| S6.1_F  S6.1_R  S6.1_P | ACACAAACATCGTGGAGTGCAC  AAGCAAATTCACCATCCCAGGC  ^FAM^CGCTGGAACACTGGAATGCC ^BHQ1^ | Position 4'285 to 4'548 (ORF2) |

Detection of neurotropic astroviruses BoAstV-CH13 & ovAstV-CH16; PanAstV-RT-PCR
