# Supplementary material for: Discovery of novel astrovirus genotype species in small ruminants
Source: PeerJ. 2019 Jul 31;7:e7338. doi: 10.7717/peerj.7338 (PMC6679648; doi:10.7717/peerj.7338)
Supplement: Supplemental Information 3 [file peerj-07-7338-s003.docx]

**Supplementary table 3: Pairwise distances of strains involved in recombination events**

Event 1 & 3

| **Identity [%] based on full-length-sequences (nucleotide level)** | | |
| --- | --- | --- |
|  | OvAstV-S5.1 | OvAstV-S6.1 |
| OvAstV-S5.1 | x | x |
| OvAstV-S6.1 | 81.8 | x |
| CapAstV-G5.1 | 77.2 | 87.6 |
| **Identity [%] based on nsp1a (amino acid level)** | | |
|  | OvAstV-S5.1 | OvAstV-S6.1 |
| OvAstV-S5.1 | x | x |
| OvAstV-S6.1 | 94.8 | x |
| CapAstV-G5.1 | 85.3 | 86.3 |
| **Identity [%] based nsp1ab (amino acid level)** | | |
|  | OvAstV-S5.1 | OvAstV-S6.1 |
| OvAstV-S5.1 | x | x |
| OvAstV-S6.1 | 94.9 | x |
| CapAstV-G5.1 | 89.8 | 90.3 |
| **Identity [%] based on capsid protein (amino acid level)** | | |
|  | OvAstV-S5.1 | OvAstV-S6.1 |
| OvAstV-S5.1 | x | x |
| OvAstV-S6.1 | 77 | x |
| CapAstV-G5.1 | 76.9 | 97.3 |

Identities are based on alignments done using the built-in MUSCLE aligner in Geneious 10.2.3.

Event 2

| **Identity [%] based on full-length-sequences (nucleotide level)** | | |
| --- | --- | --- |
|  | BoAstV/GX27 | BoAstV/JPN/HK |
| BoAstV/GX27 | x | x |
| BoAstV/JPN/HK | 55.4 | x |
| CapAstV-G2.1 | 58.6 | 70.1 |
| **Identity [%] based on nsp1a (amino acid level)** | | |
|  | BoAstV/GX27 | BoAstV/JPN/HK |
| BoAstV/GX27 | x | x |
| BoAstV/JPN/HK | 42.2 | x |
| CapAstV-G2.1 | 62.2 | 62.8 |
| **Identity [%] based nsp1ab (amino acid level)** | | |
|  | BoAstV/GX27 | BoAstV/JPN/HK |
| BoAstV/GX27 | x | x |
| BoAstV/JPN/HK | 52.4 | x |
| CapAstV-G2.1 | 64.4 | 75.4 |
| **Identity [%] based on capsid protein (amino acid level)** | | |
|  | BoAstV/GX27 | BoAstV/JPN/HK |
| BoAstV/GX27 | x | x |
| BoAstV/JPN/HK | 39.2 | x |
| CapAstV-G2.1 | 37.9 | 74.2 |

Identities are based on alignments done using the built-in MUSCLE aligner in Geneious 10.2.3.
