# Supplementary material for: Discovery of novel astrovirus genotype species in small ruminants
Source: PeerJ. 2019 Jul 31;7:e7338. doi: 10.7717/peerj.7338 (PMC6679648; doi:10.7717/peerj.7338)
Supplement: Supplemental Information 4 [file peerj-07-7338-s004.docx]

**Supplementary table 4: Overview of breakpoint positions detected by recombination analysis**

| **Event #** | **Begin** | **End** | **Recombinant Sequence(s)** | **Minor Parental Sequence(s)** | **Major Parental Sequence(s)** | **RDP** | **GENECONV** | **Bootscan** | **Maxchi** | **Chimaera** | **SiScan** | **3Seq** |
| --- | --- | --- | --- | --- | --- | --- | --- | --- | --- | --- | --- | --- |
| 1 | 4062 | 6114 | ^OvAstV-S6.1 | CapAstV-G5.1 | OvAstV-S5.1 | 4.54E-60 | 2.93E-67 | 2.86E-67 | 2.44E-10 | 2.16E-16 | 1.97E-63 | 1.11E-11 |
| 2 | 36 | 1154 | CapAstV-G2.1 | KJ620980.1  Bovine_astrovirus_strain_BAstV-GX27/CHN/2014 | LC047790.1_  Bovine_astrovirus_strain_BoAstV/JPN/Hokkaido11-55/2009 | 9.20E-55 | 5.20E-12 | 1.17E-47 | 4.94E-20 | 6.74E-24 | 4.03E-39 | 1.80E-05 |
| 3 | 1737* | 2443 | ^OvAstV-S6.1 | CapAstV-G5.1 | OvAstV-S5.1 | 1.15E-13 | 6.28E-10 | 1.31E-15 | 9.06E-08 | 1.00E-08 | 9.91E-19 | 1.11E-11 |

Key:

* = The actual breakpoint position is undetermined (it was most likely overprinted by a subsequent recombination event).

^ = The recombinant sequence may have been misidentified (one of the identified parents might be the recombinant)

Minor Parent = Parent contributing the smaller fraction of sequence.

Major Parent = Parent contributing the larger fraction of sequence.

Unknown = Only one parent and a recombinant need be in the alignment for a recombination event to be detectable.
